# Introduction of a New Pathology Workup Protocol for Glottic Cancer Treated With Transoral Laser Microsurgery (TLM): Prospective Analysis of Oncological Outcomes and Matched Case-Control Study

**DOI:** 10.3389/fonc.2021.685255

**Published:** 2021-05-04

**Authors:** Jeroen Meulemans, Sara Narimani, Esther Hauben, Sandra Nuyts, Annouschka Laenen, Pierre Delaere, Vincent Vander Poorten

**Affiliations:** ^1^ Otorhinolaryngology, Head and Neck Surgery, University Hospitals Leuven, Leuven, Belgium; ^2^ Department of Oncology, Section Head and Neck Oncology, KU Leuven, Leuven, Belgium; ^3^ Pathology, University Hospitals Leuven, Leuven, Belgium; ^4^ Department of Pathology and Imaging, KU Leuven, Leuven, Belgium; ^5^ Radiation Oncology, University Hospitals Leuven, Leuven, Belgium; ^6^ Department of Oncology, Section Experimental Radiotherapy, KU Leuven, Leuven, Belgium; ^7^ Interuniversity Institute for Biostatistics and Statistical Bioinformatics, Leuven, Belgium

**Keywords:** glottic cancer, laryngeal cancer, margins, oncological outcomes, transoral laser microsurgery

## Abstract

**Background/Purpose:**

The value of margin status after TLM for glottic cancer is debatable, due to difficulties in specimen orientation and margin analysis. To reduce these difficulties, we recently introduced a standardized protocol of oriented fixation of TLM specimens. This proved feasible and resulted in high margin evaluability rates and a decreased rate of false positive deep margins, when compared to a historical TLM cohort. For the patients whose specimens were processed according to this protocol, we prospectively analyzed oncological outcomes, identified prognostic factors and assessed the influence of the protocol introduction on outcomes compared with a historical TLM cohort.

**Methods:**

Ninety-six patients with glottic malignancies treated with TLM were included. Resection specimens were processed according to the new protocol. Descriptive statistics and survival analyses were used to determine oncological outcomes. To assess the effect of the protocol introduction on outcomes, a matched-case-control analysis was performed, using a historical TLM-cohort as controls. The Cox proportional hazards model was used to analyze prognostic effects of patient and treatment characteristics, including the pathology protocol introduction, on overall survival (OS), disease-specific survival (DSS), disease-free survival (DFS) and local recurrence-free survival (LRFS).

**Results:**

Two-year outcomes were favorable: 88.5% OS, 97.0% DSS, and 87.6% LRFS. At multivariable analysis, the presence of multiple positive superficial margins was a negative prognosticator for OS (HR 4.102) and increasing cT classification proved a negative prognosticator for DFS (HR 2.828) and LRFS (HR 2.676). Matched case-control analysis did not reveal a significant difference in oncological outcomes between cohorts. Deep margin status had a strong differential effect for DFS (p-value for interaction = 0.0205) and for LRFS (p-value for interaction = 0.0176) between cohorts, indicating a prognostic effect of deep margin status on both outcomes in the current cohort, but not in the historical cohort.

**Discussion/Conclusion:**

The introduction of a new standardized technique of oriented fixation of TLM specimens did not affect oncological outcomes when compared to a historical TLM cohort, but assigned a significant prognostic effect to deep margin status for DFS and LRFS, facilitating the decision making process with regards to planning of second-look procedures, administration of adjuvant radiotherapy or determination of follow-up intensity.

## Introduction

Over the past decades, transoral laser microsurgery (TLM) has evolved towards a well-established and highly effective minimally invasive primary treatment modality for early (cTis-cT2) and well selected cT3 glottic cancers, combining a high probability of disease control with a short hospitalization, low complication rates, good postoperative function, and excellent laryngeal preservation rates (1–8). When compared to primary external beam radiotherapy (RT), primary TLM for early glottic cancer achieves a similar survival, with lower treatment costs and a significantly higher probability of preserving the larynx (9). Moreover, in case of local recurrence, TLM leaves all salvage options open, ranging from redo-TLM over open partial laryngectomy procedures to definitive RT (10). In well-selected cases, TLM can also be considered as a potential salvage treatment for radiorecurrent glottic cancers (11). As a minimally invasive surgical technique, TLM is characterized by the concept of tumor-adapted, often piecemeal resection with implementation of ultra-narrow margins (usually 1–3 mm). The combination of ultra-narrow surgical margins on one hand and piecemeal resections, specimen orientation issues, laser coagulation artefacts at the level of the margins, and specimen shrinkage on the other hand, result in difficult and often inaccurate margin assessment (12). This is illustrated by high rates of non-evaluable or indeterminate margins after TLM, ranging from 17.2% to 33% (13) and is believed to be partially responsible for the high rates of apparently unsafe margins with reported close and positive margin rates as high as 50% (3, 14). Moreover, most close and positive margins on definitive pathology are believed to be false positive, which is illustrated by high rates of negative second-look TLM procedures (8, 15). As a result, decision making on eventually needed adjuvant therapy after TLM (e.g., second-look TLM procedure, radiotherapy) and/or follow-up intensity is complicated (10). In an attempt to optimize TLM-specimen orientation and evaluation, in order to reduce the rate of indeterminate and false positive margins, our group recently developed a new standardized protocol of orienting, inking and fixing TLM specimens on pig liver slices. In a recent publication, we proved the feasibility of this approach in both the operating room and lab setting (16). Clinical introduction of this new protocol resulted in high margin evaluability rates, especially for the deep margin (98% evaluable margins), as well as a decreased rate of false positive deep margins when compared to a historical TLM cohort. Compared to a previous series published by our group (n = 142) (15), deep margin evaluability significantly rose from 62.7% to 98.0% (p < 0.001) and true positive rate of the deep margins (determined by the discovery of residual malignancy during second-look TLM) increased from 0% to 44.4% (p = 0.002) (16). With this new protocol, we introduced a standardized way of processing the TLM specimen, leading to more accuracy in margin analysis and uniform and easy to interpret pathology reports. Together with a significantly improved reliability of the deep margin status and a significant decline in the portion of non-evaluable or indeterminate margins, this resulted in an increase in the surgeon’s confidence in the pathologic assessment, facilitating a better and easier postoperative decision-making on the need for second-look procedures, adjuvant treatment and the indicated follow-up intensity. As the initial paper focused on reporting feasibility and on the effects of the new protocol on margin evaluation, the aim of the current follow-up paper is the prospective analysis of oncological outcomes and identification of prognostic factors for outcome in the cohort of patients whose specimens were processed according to the new technique. Additionally, outcomes in the current cohort are compared by case-control matching with outcomes in a historical TLM cohort in order to examine whether the introduction of this new protocol could be an independent prognostic factor influencing oncological outcomes. Moreover, the prognostic importance of deep margin status in both cohorts is compared.

## Patients and Methods

### Study Outline

A prospective study was conducted at an academic tertiary referral hospital (University Hospitals Leuven, Leuven, Belgium) between May 2016 and September 2019. This study was approved by and carried out in accordance with the recommendations of the Institutional Review Board (University Hospital Leuven Committee for Medical Ethics, study number: S58892). Written informed consent was obtained for every included patient. All patients with glottic lesions, suspect for malignancy, who were scheduled for TLM resection of the lesion, were eligible for inclusion, including both primary as well as salvage TLM cases. The latter were patients with radiorecurrent glottic cancer or second primary glottic lesions in a previously irradiated larynx.

Our prior publication details the surgical procedure and the newly introduced pathology protocol (16). Immediately after resection, the specimens were accurately inked in the operating theatre under surgical loupe magnification using different colors to identify the different margins. After coloring the margins, the specimens were fixed with cyanoacrylate glue on a pig liver carrier, photographed, and stored in formaldehyde. They then were sent for further processing to the pathology lab. The specimen was accompanied by digital photographs of the larynx with the tumor *in situ* before resection, taken *via* the operating microscope or endoscope, and of the mounted specimen. On both photographs, the inked margins were indicated by analogous coloring, as well as areas of specific interest. A free margin was defined as a margin of ≥1 mm. The definitive pathology report of the TLM specimen, which is standardly delivered 1 week after TLM, was discussed during the multidisciplinary tumor board and the decision to submit the patient to follow-up, to a second-look procedure or to radiotherapy always resulted from a multidisciplinary discussion including the surgeon and the radiotherapist.

A second-look TLM procedure 6 to 8 weeks after the first surgery (after healing of the initial wound bed with disappearance of fibrin and completion of remucosalization) was preferentially scheduled when definitive pathologic examination suggested a deep margin and/or multiple superficial margins positive for invasive squamous cell carcinoma (SCC). A “true positive margin” was defined as a positive margin (deep or superficial) on initial pathologic examination which was confirmed by the persistent presence of invasive SCC in the resection specimen of the wound bed obtained during second-look TLM.

Administration of radiotherapy was only considered when (1) surgical margins after a second-look TLM procedure, performed for margin positivity after initial TLM, were again considered compromised or (2) when dealing with very infiltrative tumors difficult to delineate during surgery and resulting in deep and multiple superficial margin positivity after initial TLM in combination with the surgeon’s estimate of a low probability of achieving free margins with a second-look procedure.

Postoperative follow-up visits with flexible nasolaryngoscopy including narrow-band imaging (NBI) were organized every 2 months during the first 2 years, every 3 months during the 3rd year, every 4 months during the 4th year and every 6 months thereafter (17). Baseline imaging of the neck (CT or MRI) was performed 3 to 4 months after treatment and was repeated 1 and 2 years after treatment to exclude submucosal locoregional recurrence in more high-risk cases (T2 with impaired vocal fold mobility-T3) (18). As opposed to the prior feasibility study (n = 104) (16), patients whose glottic lesions proved benign on definitive pathological examination were excluded *post hoc*. Moreover, while the feasibility study allowed for multiple entries of a same patient in the study (1 entry for every TLM procedure), multiple entries were reduced to the first TLM procedure in this follow-up study. As such, the resulting patient cohort consists of different individual patients who underwent TLM for proven (pre)malignant glottic lesions (n = 96).

### Data Collection and Variable Description

Data were entered into an individual electronic case report file (eCRF) (Access 2016, Microsoft, Redmond, USA) and later transferred into a database gathering all data of included patients (SPSS PC version 22.0, IBM Corp, Armonk, NY, USA). Prospectively registered data were related to the patient (age, gender, smoking status, ethyl use), the tumor (cT/cN/pT classification according to UICC 8th edition tumor staging manual (19), involvement of the anterior commissure (AC), tumoral extension into the subglottic, and/or supraglottic region) and the pathological evaluation (worst histology in the specimen, superficial and deep margin status for invasive component, presence of carcinoma *in situ* (CIS) or dysplasia in superficial margins, and presence of multiple positive superficial margins for CIS or invasive SCC). Information on surgery, postoperative course, and adjuvant treatment included setting (primary versus salvage), the primary therapy in salvage cases, type of cordectomy performed according to the ELS classification (20, 21), postoperative complications, duration of hospitalization, second-look procedures with the resulting histology, and administration of adjuvant RT. Finally, data related to oncological outcomes were registered, such as development of recurrence during follow-up (FU), type of recurrence (local/regional/locoregional), time interval between TLM and recurrence, treatment modality of first and subsequent recurrences, total number of TLM procedures, development/location/treatment of a second primary tumor, date of last FU, occurrence of death and its cause, and lastly, necessity and reason for total laryngectomy.

### Definitions of Oncological Outcome Measures and Statistical Methodology

For all oncological outcome measures, the starting point is the date of the first TLM procedure. Overall survival (OS) is defined as the time between the first TLM procedure and death of any cause. Patients alive are censored at last follow-up. Disease-specific survival (DSS) is the time between the first TLM procedure and disease-related death. Non–disease-related death is considered a competing event. Patients alive and disease-free are censored at last follow-up. Disease-free survival (DFS) is the time between first TLM and the earliest among recurrence (local, regional or distant) or disease-related death. Non–disease-related death is considered a competing event. Patients alive and disease-free are censored at last follow-up. Local relapse- or recurrence-free survival (LRFS) is the time between first TLM and the earliest among local recurrence or disease-related death. Non disease-related death is considered a competing event. Patients alive and local relapse-free are censored at last follow-up.

Ultimate local control (ULC) by TLM alone is defined as the time between TLM and the earliest among adjuvant RT, local recurrence treated otherwise than by TLM or disease-related death. Non–disease-related death is considered a competing event. Patients alive and event-free are censored at last follow-up. The Kaplan-Meier method was used for estimating OS. The cumulative incidence function approach was used for DSS, DFS, LRFS, and ULC by TLM alone accounting for non-disease related death as competing event and for larynx preservation rate, accounting for death as competing event. For the analysis of the current database, the Cox proportional hazards model was used to analyze prognostic effects of patient- or treatment-characteristics on OS, DSS, DFS, and LRFS. Results are presented as hazard ratios (HR) with 95% confidence intervals. A forward selection procedure was used for the selection of a multivariable model of independent prognostic variables for oncological outcomes, with a 5% significance level for entering of variables. A matched-case-control study was conducted by comparing our current cohort with the implemented new pathology protocol (cases), to a matched sample of controls, retrieved from a historical TLM cohort, previously published by our group (15). Propensity score matching was performed for matching cases to controls in a 1:1 ratio (22, 23). Data analysis was performed taking into account clustering by matching. Cox models with robust variance estimator were used for time-to-event outcomes, and conditional logistic regression for binary outcomes. All tests are two-sided, and a 5% significance level was assumed. To assess whether the prognostic effect of deep margin status on oncological outcomes was different between both cohorts, deep margin status was analyzed as a binary variable (positive versus free/close). Cases with non-evaluable margins were excluded for this analysis. An interaction term was used between both variables (deep margin status [positive versus free/close] and cohort type [historical versus current]), to test whether there was statistical evidence for a differential effect of deep margin status in both cohorts.

Analyses have been performed using SAS software (version 9.4 of the SAS System for Windows).

## Results

### Patient, Tumor, and Treatment Characteristics

We included 96 patients (86 males, 10 females) in this prospective outcome study. Tumors were pre-operatively staged as cT1a (n = 62; 64.6%), cT1b (n = 13; 13.5%), cT2 (n = 20; 20.8%), and cT3 (n = 1; 1.0%). Intraoperatively, extension of the tumor into the anterior commissure (AC) was apparent in 38 cases (39.6%). Involvement of the subglottic and supraglottic area was seen in 16 (16.7%) and nine (9.4%) patients, respectively. All patients were cN0. Eighty-seven patients (90.6%) underwent primary TLM, nine patients (9.4%) were treated in a salvage setting. Salvage patients had been primarily treated with RT (n = 8) or radiochemotherapy (n = 1). Mean and median time interval between primary RT or CRT and salvage TLM were 39 and 20 months, respectively. Type of TLM procedures performed were cordectomy type I (n = 21; 21.9%), II (n = 24; 25.0%), III (n = 28; 29.2%), Va (n = 15; 15.6%), Vb (n = 3; 3.1%), Vd (n = 2; 2.1%), and VI (n = 3; 3.1%). Mean and median postoperative hospitalization duration was 1.7 and 1 days, respectively (interquartile range 1.0–2.0 days and range 1.0–18.0 days). Complications occurred in nine patients (9.4% of the population) and consisted of postoperative hemorrhage (n = 1), dysphagia and/or aspiration (n = 5) and limited chondronecrosis of the thyroid cartilage (n = 3). In general, 18 second-look procedures were performed, yielding residual invasive SCC in five cases (27.8%). Mean and median time intervals between initial TLM procedures and second-look procedures were 57.6 and 56 days, respectively. According to our institutional policy, most patients with a deep margin and/or multiple superficial margins positive for invasive SCC were scheduled for a second-look TLM (n = 4 out of 5 patients with a positive deep margin, n = 2 out of three patients with multiple positive superficial margins and n = 5 out of 7 patients with combined positive deep and multiple superficial margins). Other reasons for performing a second-look TLM were: a deep margin non-evaluable at pathological examination (n = 1), presence of a close deep margin (n = 2) and subjective preference of the treating surgeon (e.g. intra-operative doubt about resection radicality (n = 4). In the nine patients with involved deep margins who underwent a second-look procedure, residual invasive SCC was found in four specimens (44.4%; three primary cases and one salvage case) with the resection considered adequate and the patients submitted to postoperative follow-up in three cases. One patient was eventually scheduled for definitive radiotherapy because margins were again considered compromised. Moreover, for two patients, definitive radiotherapy was preferred above second-look TLM because of deep and multiple superficial margin positivity in combination with the surgeon’s estimate of a low probability of achieving free margins with a second-look procedure. As such, three patients received adjuvant radiotherapy (3.1%) with a median time-interval between initial TLM procedures and start of radiotherapy of 33 days. More data about patient, tumor and treatment characteristics are depicted in **Tables 1** and **2**.

**Table 1:** Overview of patient and tumor characteristics.

Variable	Value	Proportion (%)
**Age (years)**		
Mean	69.02	
SD	10.972	
**Gender**		
Male	86/96	88.58
Female	10/96	10.42
**Smoker**		
No	10/93	10.75
Past smoker	62/93	66.67
Active smoker	21/93	22.58
**Number of packyears**		
Mean	38.42	
SD	20.685	
**Active ethyl**		
No	22/90	24.44
Yes	68/90	75.56
**Ethyl Units/week**		
Mean	14.11	
SD	19.225	
**cT**		
T1a	62/96	64.58
T1b	13/96	13.54
T2	20/96	20.83
T3	1/96	1.04
**cN**		
N0	96/96	100.00
**pT**		
Tis	36/96	37.50
T1a	37/96	38.54
T1b	5/96	5.21
T2	16/96	16.67
T3	2/96	2.08
**Anterior commissure involvement**		
No	58/96	60.42
Yes	38/96	39.58
**Subglottic extension**		
No	80/96	83.33
Yes	16/96	16.67
**Supraglottic extension**		
No	87/96	90.63
Yes	9/96	9.38

IQR, interquartile range.

**Table 2:** Overview of treatment characteristics.

Variable	Value	Proportion (%)
**Primary/salvage**		
Primary TLM	87/96	90.63
Salvage TLM for recurrence after RT	9/96	9.38
**Primary treatment in salvage cases**		
RT	8/9	88.89
Radiochemotherapy	1/9	11.11
**ELS classification of cordectomy**		
Type I	21/96	21.88
Type II	24/96	25.00
Type III	28/96	29.17
Type Va	15/96	15.63
Type Vb	3/96	3.13
Type Vd	2/96	2.08
Type VI	3/96	3.13
**Postoperative complications**		
Bleeding	1/9	11.11
Dysphagia	1/9	11.11
Aspiration	4/9	44.44
Chondronecrosis	3/9	33.33
**Hospitalization duration (days)**		
Mean	1.66	
SD	1.999	
Median	1.00	
IQR	1.00;2.00	
Range	1.00–18.00	
**Second-look**		
No	78/96	81.25
Yes	18/96	18.75
**Histology of second-look**		
No residual malignancy	12/18	66.67
Dysplasia	1/18	5.56
Invasive SCC	5/18	27.78
**Adjuvant RT**		
No	93/96	96.88
Yes	3/96	3.13

### Pathology Results

The worst histology encountered in the specimen during pathologic evaluation was low- and high-grade dysplasia in seven (7.3%) and eight (8.3%) cases, respectively, CIS in 21 (21.9%) cases and invasive SCC in 60 (62.5%) specimens. The deep margin was free from invasive SCC in 62 cases (66.0%), close in 18 cases (19.2%), positive in 12 cases (12.8%) and non-evaluable in only two patients (2.1%). Ten patients (10.4%) exhibited multiple superficial margin positivity (for invasive SCC).

### Oncological Outcomes and Prognostic Factors

**Figure 1:**
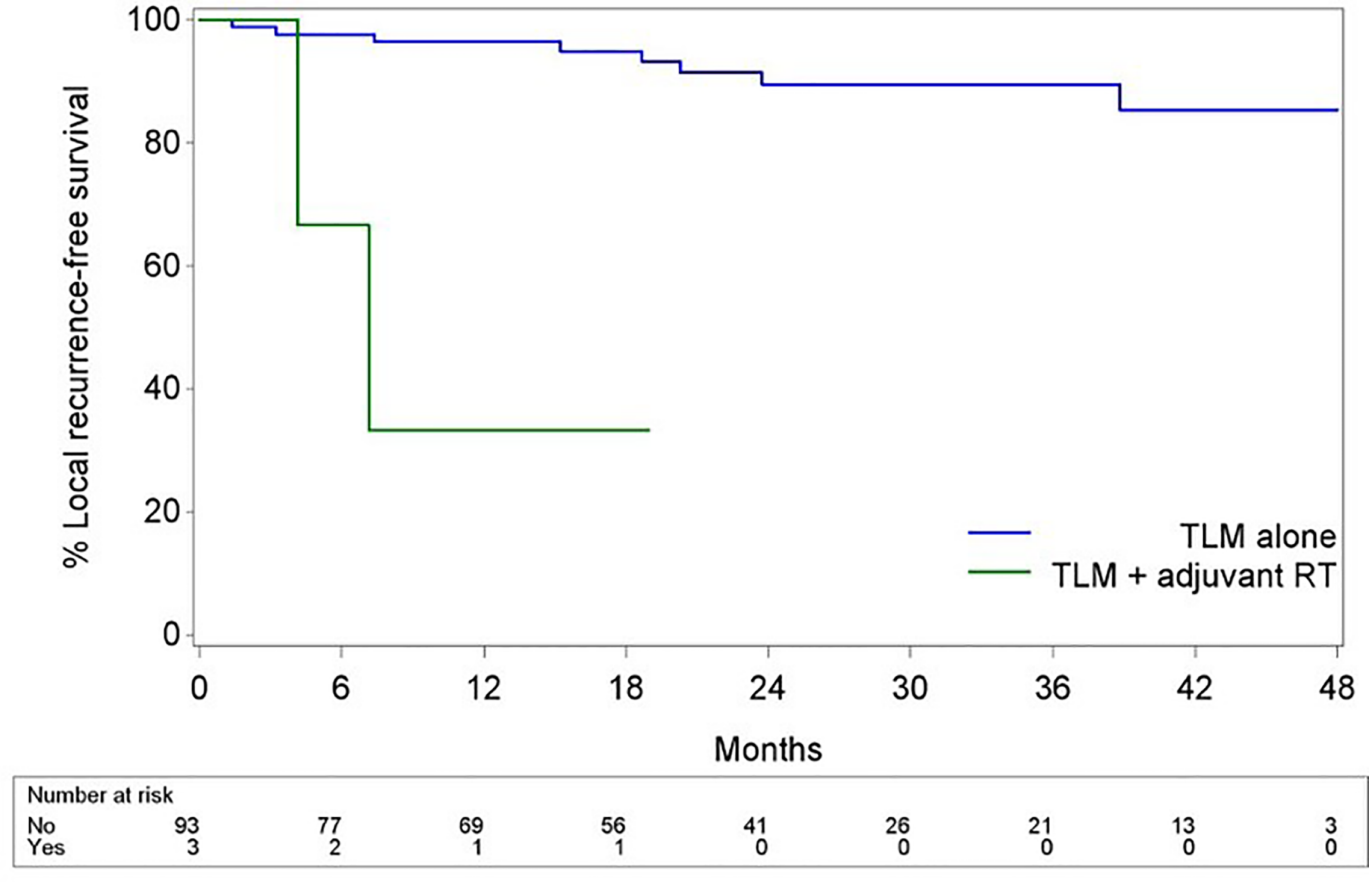
Kaplan-Meier curves illustrating LRFS with TLM alone vs LRFS with TLM + adjuvant RT. Subjects who received adjuvant RT showed significantly poorer LRFS when compared to subjects treated with TLM alone (HR 15.974, p = 0.001).

Mean and median follow-up after the first TLM procedure were 23.6 and 22.4 months respectively. For the patients alive at last follow-up (FU), mean and median follow-up were both 24.5 months. During FU, 11 patients (11.5%) developed disease recurrence of whom 10 (90.9%) recurred locally and 1 (9.1%) locoregionally. Four recurrences were salvaged with TLM (36.4%), two with TLM and RT (18.2%), four with total laryngectomy (36.4%) and one with RT and chemotherapy (9.0%). One patient developed a second local recurrence and was again salvaged with TLM. Eventually, ultimate local disease control at the end of follow-up exclusively obtained with TLM procedures was achieved in 89 subjects (92.7%). Second primary tumors developed in 10 patients (10.4%) of which three were located in the upper aerodigestive tract (floor of mouth). A total laryngectomy proved necessary in five cases (5.2%), either for disease recurrence (n = 4, 80.0%) or for intractable aspiration (n = 1, 20.0%), resulting in an overall laryngeal preservation rate at the end of follow-up of 94.8%. Laryngeal preservation rate with TLM alone, defined as the rate of patients who had, at the end of follow-up, a preserved larynx and were exclusively treated with TLM (as a first treatment and for recurrence if applicable) was 90.6%. During follow-up, nine patients (9.4%) died, with three deaths being related to the primary laryngeal cancer. Estimates for OS, DSS, DFS, LRFS, ultimate local control with TLM alone and laryngeal preservation rate at 24 and 48 months are summarized in **Table 3**. Of interest, when comparing the subgroup of patients treated for their first tumor with TLM only versus the subset of patients treated with TLM and adjuvant radiotherapy (n = 3), the 2- year estimate of LRFS was significantly lower in the TLM+RT group (33.3% versus 89.5%, HR = 15.974, p = 0.0011) (**Figure 1**). Indeed, out of the three patients who were treated with TLM+RT, two recurred locally, of whom one was salvaged by TLM and one who eventually died of disease after aggressive disease recurrence following salvage total laryngectomy.

**Table 3:** Estimates for OS, DSS, DFS, LRFS, ULC with TLM alone and laryngeal preservation rate.

Variable	Survival estimates % (95% CI)
***Overall survival***	
24 months	88.47 (77.97–94.15)
48 months	86.20 (74.65–92.74)
***Disease-specific survival***	
24 months	97.01 (90.73–99.44)
48 months	94.75 (86.58–98.67)
***Disease-free survival***	
24 months	86.24 (77.06–93.08)
48 months	82.22 (70.22–91.48)
***Local recurrence-free survival***	
24 months	87.58 (78.64–94.03)
48 months	83.62 (71.75–92.48)
***Ultimate local control with TLM alone***	
24 months	92.47 (85.19–96.98)
48 months	88.38 (76.86–95.78)
***Laryngeal preservation rate***	
24 months	93.29 (85.96–97.57)
48 months	93.29 (85.96–97.57)

At univariable analysis, increasing clinical and pathological tumor classification (cT/pT), subglottic extension of the tumor, a positive deep margin, and multiple superficial margin positivity for invasive SCC all were identified as negative prognostic factors influencing various oncological outcome parameters parameters (**Table 4**). However, at multivariable analysis, only multiple superficial margin positivity for invasive SCC could be confirmed as an independent negative prognostic factor for OS, and increasing cT-classification proved an independent negative prognosticator for DFS, LRFS and LRFS with TLM alone (exclusion of cases treated with TLM+adjuvant RT). Continuation of smoking after the first TLM procedure, treatment setting (primary versus salvage), anterior commissure involvement, extension of the tumor in the supraglottic region, worst histology in the specimen (dysplasia versus CIS versus invasive SCC) and presence of CIS in the superficial margins did not have a significant impact on any of the oncological outcomes, neither in univariable analysis nor in multivariable analysis. As only three events were identified for DSS, no uni- or multivariable analyses could be performed for DSS. Additionally, we assessed the prognostic effect of adjuvant treatment with RT on oncological outcomes in the subgroup of patients with multiple positive superficial and/or positive deep margins (= patients at high risk for local recurrence). In this subgroup of 15 patients, three received adjuvant RT; all had cT2 tumors with deep margins positive for invasive SCC and two of these also additionally had multiple superficial margin positivity for invasive SCC. In contrast, the group who did not receive adjuvant RT but only second-look TLM included four cT2 and eight cT1a patients with, at the 1st TLM procedure, 33.3% (n = 4) of patients having positive deep margins, 25% (n = 3) having multiple positive superficial margins and 41.7% (n = 5) having both involved deep and multiple superficial margins. At univariable analysis, the need to administer adjuvant RT proved an indication that the patient belonged to a prognostically unfavorable subgroup, with adjuvant RT identified as a significant negative prognosticator for DFS (HR 11.866, p = 0.0461) and LRFS (HR 11.866, p = 0.0461). Moreover, a trend towards poorer OS was seen in the adjuvant RT group (HR 10.474, p = 0.0566).

**Table 4:** Overview of significant prognosticators for various oncological outcomes after TLM, as identified at univariable and multivariable analyses.

Variable	Univariable identification of prognostic value of variable for oncological outcomes	Multivariable confirmation of prognostic value of variable for oncological outcomes
pT*	**OS** (HR 1.683, p = 0.0494)	
	**DFS** (HR 1.769, p = 0.0149)	
	**LRFS** (HR 1.654, p = 0.0409)	
cT*	**DFS** (HR 2.828, p = 0.0013)	**DFS** (HR 2.828, p = 0.0013)
	**LRFS** (HR 2.676, p = 0.0034)	**LRFS** (HR 2.676, p = 0.0034)
	**LRFS with laser alone** (HR 2.394, p = 0.016)	**LRFS with laser alone** (HR 2.394, p = 0.0160)
***Subglottic extension***	**DFS** (HR 3.756, p = 0.0290)	
	**LRFS with laser alone** (HR 4.413, p = 0.036)	
***Multiple superficial margin positivity for***	**OS** (HR 4.102, p = 0.047)	**OS** (HR 4.102, p = 0.047)
***invasive SCC***	**DFS** (HR 4.102, p = 0.0467)	
	**LRFS** (HR 4.102, p = 0.0467)	
	**LRFS with laser alone** (HR 4.102, p = 0.0467)	
***Deep margin positivity for invasive SCC***	**DFS** (HR 5.059, p = 0.0110) **LRFS** (HR 6.425, p = 0.0061)	

*cT and pT were analyzed as ordinal variables. Indicated HR is related to increase of cT and pT with one level.

OS, overall survival; DSS, disease specific survival; DFS, disease free survival; LRFS, local relapse/recurrence free survival; SCC, squamous cell carcinoma.

### Matched Case-Control Study and Differential Prognostic Effect of Deep Margin Status

In order to assess the effect of the introduction of the new pathology protocol on oncological outcomes, cases included in the current cohort were matched to historical controls for age, gender, treatment setting (primary versus salvage), cT-classification, UICC tumor stage, administration of adjuvant RT, worst histology in the specimen (CIS versus invasive SCC) and ELS type of cordectomy. After exclusion of patients with dysplasia as worst histology from the current cohort (because there were no dysplasia cases in historical cohort) and exclusion of patients with missing data on matching variables from the historical cohort, 79 cases could be matched with controls (**Table 5**). After matching, no significant differences in OS (HR 1.587, p = 0.1780), DSS (HR 3.156, p = 0.1338), DFS (HR 0.941, p = 0.8964), LRFS (HR 0.948, p = 0.9149), laryngeal preservation rate (OR 0.750, p = 0.7064) and laryngeal preservation rate with TLM alone (OR 1.572, p = 0.3499) between both cohorts were observed (higher probability of event in current cohort compared to historical if OR/HR>1). To account for potential overmatching, a re-analysis was performed with adjuvant RT excluded as a matching variable. After all, introduction of the new pathology protocol and the resulting improved margin assessment influenced decision-making regarding adjuvant treatment. However, this re-analysis did not result in different findings.

**Table 5:** Overview of matching variables for current and historical cohort: patient, tumor and treatment characteristics.

Variables	Current (n = 79)	Historical (n = 79)
***Age (years)***		
Mean	68.57	67.80
SD	11.822	12.100
Range	(27.96; 94.04)	(35.36; 89.55)
***Gender***		
Male	71/79 (89.87%)	71/79 (89.87%)
Female	8/79 (10.13%)	8/79 (10.13%)
***Primary/salvage***		
Primary TLM	70/79 (88.61%)	68/79 (86.08%)
Salvage TLM	09/79 (11.39%)	11/79 (13.92%)
***cT***		
T1a	51/79 (64.56%)	52/79 (65.82%)
T1b	9/79 (11.39%)	7/79 (8.86%)
T2	18/79 (22.78%)	19/79 (24.05%)
T3	1/79 (1.27%)	1/79 (1.27%)
***Tumor stage***		
Stage 0	21/79 (26.58%)	10/79 (12.66%)
Stage I	41/79 (51.90%)	57/79 (72.15%)
Stage II	15/79 (18.99%)	11/79 (13.92%)
Stage III	2/79 (2.53%)	1/79 (1.27%)
***Adjuvant RT***		
No	76/79 (96.20%)	75/79 (94.94%)
Yes	3/79 (3.80%)	4/79 (5.06%)
***Worst histology***		
Invasive SCC	58/79 (73.42%)	57/79 (72.15%)
CIS	21/79 (26.58%)	22/79 (27.85%)
***ELS classification of cordectomy***		
Type I	13/79 (16.46%)	14/79 (17.72%)
Type II	19/79 (24.05%)	15/79 (18.99%)
Type III	27/79 (34.18%)	30/79 (37.97%)
Type IV	0/79 (0.00%)	2/79 (2.53%)
Type Va	13/79 (16.46%)	8/79 (10.13%)
Type Vb	3/79 (3.80%)	1/79 (1.27%)
Type Vc	0/79 (0.00%)	3/79 (3.80%)
Type Vd	2/79 (2.53%)	1/79 (1.27%)
Type VI	2/79 (2.53%)	5/79 (6.33%)

As we reported in our previous publication, the introduction of the new standardized pathology workup protocol led to a significant increase in deep margin evaluability rate when compared to the historical cohort (98.0% versus 62.7%, p<0.001) (16). As a consequence, a large proportion of controls from the historical cohort had a non-evaluable deep margin status in the original case-control matching. To avoid loss of information, a separate matching was performed for the analysis of the differential effect of deep margin status, based on the same principles but using only historical controls with non-missing deep margin status, resulting in 65 case-control matches with known deep margin status. In the current cohort, significantly more deep margins were considered free (61.5% versus 23.1%, p<0.001) while of patients with evaluable deep margins in the historical cohort, a significantly larger proportion of deep margins was considered positive (66.2% versus 15.4%, p<0.001). When the prognostic effect of deep margin status (positive versus free/close) on oncological outcomes in both cohorts was compared (‘differential prognostic effect’ of deep margin status), no difference on OS could be identified (p-value for interaction = 0.5406). However, deep margin status proved to have a significantly differential effect on DFS (p-value for interaction = 0.0205) and LRFS (p-value for interaction = 0.0176), indicating a strong negative prognostic effect of positive deep margin status on both outcomes in the current cohort, but not in the historical cohort (DFS: HR 6.200, p 0.0056 in current cohort versus HR 0.802, p 0.6847 in historical cohort; LRFS: HR 8.304, p 0.0033 in current cohort versus HR 0.809, p 0.7285 in historical cohort).

## Discussion

Transoral laser microsurgery (TLM) results in reported 10- and 20-year DSS rates of 97.6% and 96.3%, respectively, and 10- and 20-year organ preservation rates of 94.7% and 93% respectively (7). These numbers result from studies with a retrospective design, but are confirmed in our prospective study. In 96 consecutive patients, we report a 2-year OS of 88.5%, 2-year DSS of 97.0%, 2-year DFS of 86.2%, 2-year LRFS of 87.6%, 2-year ULC with TLM alone of 92.5% and 2-year organ/laryngeal preservation rate of 93.3%.

In our study, a “positive deep margin” and “multiple positive superficial margins” were both identified at univariable analysis as negative prognostic factors influencing various oncological outcome parameters, with “multiple superficial margin positivity” remaining significant in multivariable analysis. The prognostic value of margin status has previously been reported: in a retrospective study including 590 patients with cTis-cT3 glottic cancer who underwent TLM with curative intent, close/positive margin status was identified as a negative prognostic factor for OS and larynx-preservation rate at multivariable analysis (8). Fiz et al. illustrated that all types of margin positivity predict the occurrence of relapses, albeit with different likelihood, depending on pT-status of the tumor and the type of the margin; in pTis–T1b patients, DSS and RFS were reduced in patients with multiple positive superficial and positive deep margins. In pT2 patients, DSS was reduced in multiple positive superficial margins and RFS was reduced in single positive superficial, multiple positive superficial, and positive deep margins. In the entire population, RFS was reduced in close deep margins (24). In our series, multiple superficial margin positivity was identified as an independent negative prognostic factor for OS (HR 4.102, p = 0.0047) at multivariable analysis and additionally for DFS (HR 4.102, p = 0.0467), LRFS (HR 4.102, p = 0.0467) and LRFS with laser alone (HR 4.102, p= 0.0467) at univariable analysis. Deep margin positivity however did not show an independent prognostic effect at multivariable analysis, but could be identified as a negative prognosticator for DFS (HR 5.059, p = 0.0110) and LRFS (HR 6.425, p = 0.0061) at univariable analysis. Due to the very low number of events and the subsequent low power to perform a multivariable analysis in our rather small study population, the results of the multivariable analysis need to be interpreted with caution and the variables identified as having prognostic significance (e.g. deep margin status) at univariable analysis should not be neglected. The aforementioned findings positively validates our strategy of favoring a second-look TLM procedure with resection of the initial wound bed for these patients with positive deep margins and/or multiple mucosal/superficial margins positive for invasive SCC.

Apart from margin status, T-classification is a well-known factor influencing oncological outcomes after TLM. In our series, cT-classification was identified as a multivariably independent prognostic factor for DFS (HR 2.828, p = 0.0013), LRFS (HR 2.676, p = 0.0034) and LRFS with laser alone (HR 2.394, p = 0.0160), with increasing cT-classification implying worse outcomes. Pathological T-classification was identified as a negative prognostic factor for OS (HR 1.683, p = 0.0494), DFS (HR 1.769, p = 0.0149) and LRFS (HR 1.654, p = 0.0409) at univariable analysis, but lost its significance in a multivariable model containing cT. In the series by Ansarin et al, pT-classification influenced RFS and larynx-preservation rate at multivariable analysis, with pTis patients having poorer RFS compared to pT1 patients and similar RFS compared to pT2-pT3 patients at one hand and better organ preservation rates compared to pT2 patients on the other (8). Within pT1 patients, pT1a lesions showed better RFS compared to pT1b lesions (24). Moreover, pT-classification has been shown to have a significant impact on DSS (25). Although T-classification is a significant prognosticator for oncological outcomes, the TNM staging system is considered too simplistic to precisely define the different possible extensions of glottic tumors, as profound differences exist among tumors usually grouped together under the same T-category but presenting a very different oncological prognosis. Piazza et al. introduced an interesting concept of a three-dimensional map of isoprognostic zones in glottic SCC, which is more accurately predicting outcomes than T-classification. In particular, pT2 lesions extending superficially to the supraglottis or subglottis had a significant better prognosis than pT2 lesions infiltrating the vocal muscle. Moreover, pT3 lesions involving the anterior paraglottic space and pT2 or pT3 tumors with vertical extension across the AC showed a significantly increased risk of local recurrence and a significantly reduced probability to achieve local control with laser alone and organ preservation (26).

Extension of the tumor to the subglottic area was identified in our univariable analysis as a negative prognosticator for DFS (HR 3.756, p = 0.0290) and LRFS with laser alone (HR 4.413, p = 0.036), but this could not be confirmed as an independent prognostic factor at multivariable analysis. The prognostic value of subglottic extension of a glottis tumor was illustrated by Carta et al., who observed at multivariable analysis a statistically worse RFS, local control with laser alone and overall laryngeal preservation rate in T2 patients with a true subglottic spread (defined as more than 1 cm below the glottic plane) despite free surgical margins (74, 67.3, and 84.6%, respectively, vs. 85.6, 85.6, and 90%, respectively, observed in T2 without subglottic extension) (27).

Although our series confirmed the prognostic effect of T-classification, margins status and subglottic spread on outcomes, the previously reported prognostic effect of ELS-type of cordectomy and anterior commissure involvement could not be confirmed (8, 28, 29).

Of particular interest in our series is the apparent negative effect of adjuvant radiotherapy on outcomes. When comparing the subgroup of patients treated for their first tumor by TLM only versus the subset of patients treated by TLM and adjuvant radiotherapy (n = 3), the 2- year estimate of LRFS was significantly lower in the TLM+RT group (33.3% versus 89.5%, HR = 15.974, p = 0.0011). Moreover, in the subgroup of patients with multiple positive superficial and/or positive deep margins, patients who were referred for RT based on the clinical judgement of the poor probability of achieving radical tumor clearance by redo TLM did significantly worse. This was illustrated by identification of adjuvant RT need as a negative prognosticator for DFS and LRFS (both HR 11.866, p = 0.0461) at univariable analysis. Of course, as adjuvant radiotherapy is reserved for the most aggressive lesions, selection bias is a likely explanation. Moreover, the small number of patients who received adjuvant RT necessitates interpreting these findings with caution. On the other hand, this may indicate that, when being faced with negative prognostic factors upon pathological examination (positive deep margins and/or multiple positive superficial margins), the administration of adjuvant RT seems to fail in ameliorating the outcome of these high-risk patients. In line with this finding, Ansarin et al. observed a similar 5-year local recurrence rate in a group of patients with close to positive margins not subjected to further treatments in comparison to a group with close-to-positive margins subjected to RT (8). Additionally, in the recent retrospective case series by Piazza et al., patients salvaged by (chemo)radiotherapy for a local recurrence after primary TLM had a significantly higher chance of dying of disease (p = 0.008; HR 6.6) when compared to patients selected for open partial horizontal laryngectomies (OPHL’s) and repeat TLM procedures, with OPHL implying a higher chance of eventually needing a total laryngectomy (p = 0.047; HR 3.5) when compared with (chemo)radiotherapy and redo TLM. As such, patients amenable to redo TLM had optimal results in terms of DSS and organ preservation rates. When taking the evidence mentioned above into account, second-look TLM procedures for involved margins and redo TLM procedures for local recurrences after failure of primary TLM seem to provide optimal oncological results in patients for whom an experienced surgeon judges this procedure to have a reasonable chance of being successful. Piazza et al. suggest that, in recurrent cases not manageable by salvage TLM, OPHL, although associated with lower laryngeal preservation, may result in a better DSS than RT. However, in interpreting this observation, a potential bias of patient selection should be taken into account, since patients with more severe comorbidities (e.g. poor pulmonary function) are more frequently directed to RT (7). As such, when dealing with involved margins (especially positive deep and/or multiple mucosal/superficial margins) after initial TLM, achievement of definitive free margins by performing a second-look TLM procedure is absolutely preferred. However, when a second-look TLM is judged very unlikely to result in “true negative margins” (e.g. tumor spread in the posterior paraglottic space), or when a second-look procedure yields again several foci of residual disease or positive margins, adjuvant therapy is necessary and could include RT or OPHL. In experienced hands, OPHL is an efficient strategy to achieve local tumor control after TLM failure (30, 31). Moreover, OPHL keeps all salvage options (radiotherapy, total laryngectomy) open in case of recurrence. However, it is also important to consider that the success of OPHL in achieving local control combined with favorable functional outcomes is highly dependent of the treating team’s experience with this demanding surgical procedure. Especially in centers which don’t offer OPHL, radiotherapy is favored in the adjuvant setting following initial TLM.

The introduction of the new standardized technique of oriented fixation of TLM specimens on a pig liver carrier resulted in a significant rise in deep margin evaluability rate from 62.7% in a historical TLM cohort to 98.0% (p<0.001). Moreover, the true positive rate of the deep margins increased from 0% to 44.4% (p = 0.002) (16). Although, when compared to a historical cohort, we did not observe superior oncological outcomes in the cohort that was evaluated with the new pathology protocol, we did see that deep margin status got a stronger prognostic effect on DFS and LRFS. We hypothesize that in the historical cohort, many ‘positive deep margins’ were in fact false positives (0% residual SCC upon second-look TLM), which might have hampered the observation of a prognostic effect of deep margin status in the historical cohort. As such, the introduction of the new pathology protocol assigns a significant negative prognostic effect of positive deep margins status on DFS and LRFS, which facilitates the decision making process with regards to planning of second-look procedures, administration of adjuvant radiotherapy or choice of follow-up intensity. However, this finding needs to be interpreted with caution, as positive deep margin status could not be identified as an independent prognosticator for outcome at multivariable analysis in the global current population. As false positive margins after TLM are likely to result in overtreatment (e.g. unnecessary second-look TLM procedures, adjuvant RT), the reduction in false positive margins could potentially result in a reduction in overtreatment. The non-inferiority of oncological outcomes in the historical cohort could partially be explained by the fact that, apart from the margin status on pathologic examination, the intra-operative opinion of the experienced TLM surgeon on resection radicality, especially for the superficial margins, is an important factor in decision-making concerning second-look procedures or adjuvant therapy. With the identification of multiple superficial margin positivity as an independent negative prognostic factor for OS at multivariable analysis, and the assignment of a prognostic effect of deep margin status for DFS and LRFS upon matched case-control analysis, our current practice of performing second-look TLM procedures in cases of positive deep margins and/or multiple positive superficial margins has been confirmed as a sensible strategy. With the introduction of this easy, time-efficient and standardized pathology approach, the former “gut-feeling” of the experienced TLM surgeon concerning surgical radicality gets more objective and standardized backup, which is of great value during the postoperative multidisciplinary decision making process.

## Conclusion

The introduction of a new standardized technique of oriented fixation of TLM specimens on pig-liver slices did not affect oncological outcomes when compared to a historical TLM cohort. However, it assigns a significant prognostic effect for DFS and LRFS to deep margin status, which facilitates the decision making process with regards to planning of second-look procedures, administration of adjuvant radiotherapy or determining follow-up intensity.

## Data Availability Statement

The raw data supporting the conclusions of this article will be made available by the authors, without undue reservation.

## Ethics Statement

The studies involving human participants were reviewed and approved by University Hospital Leuven Committee for Medical Ethics. The patients/participants provided their written informed consent to participate in this study.

## Author Contributions

JM and VP contributed to study setup, data collection, data quality control, data analysis (statistics), drafting manuscript, and review of manuscript. EH contributed to study setup, data collection, drafting manuscript, and review of manuscript. SNa contributed to data collection, drafting manuscript, and review of manuscript. AL contributed to data quality control, analysis (statistics), drafting manuscript, and review of manuscript. SNu and PD contributed to drafting manuscript and review of manuscript. All authors contributed to the article and approved the submitted version.

## Funding

Costs related to statistical analysis and manuscript publication were funded through the Vandeputte Walter Hoofd-Halskanker fund of the KU Leuven.

## Conflict of Interest

The authors declare that the research was conducted in the absence of any commercial or financial relationships that could be construed as a potential conflict of interest.
